# Surface-Degradable Drug-Eluting Stent with Anticoagulation, Antiproliferation, and Endothelialization Functions

**DOI:** 10.3390/biom9020069

**Published:** 2019-02-18

**Authors:** Ruixia Hou, Leigang Wu, Jin Wang, Zhilu Yang, Qiufen Tu, Xingcai Zhang, Nan Huang

**Affiliations:** 1 Department of Anatomy and Histology and Embryology, Medical School of Ningbo University, Ningbo 315211, China; 2 Key Laboratory of Advanced Technology of Materials of Education Ministry, School of Materials Science and Engineering, Southwest Jiaotong University, Chengdu 610031, China; gangleiwu@163.com (L.W.); wangjin@swjtu.edu.cn (J.W.); zhiluyang1029@swjtu.edu.cn (Z.Y.); tuqiufen@swjtu.edu.cn (Q.T.); 3 John A. Paulson School of Engineering and Applied Sciences, Harvard University, Cambridge, MA 02138, USA

**Keywords:** stent coatings, surface erosion, drug carrier, rapamycin, smooth muscle cells

## Abstract

Drug-eluting stents (DES) have been widely applied for saving the life of patients with coronary artery diseases (CADs). However, conventional polymers such as polylactic acid (PLA) and poly (lactic-co-glycolic acid) (PLGA), which are widely applied for drug-eluting stents studies, have serious bulk erosion problems, like high local acidity and poor mechanical properties. Instead, we chose surface erosion polymer poly (1, 3-trimethylene carbonate) (PTMC) as a drug carrier in this study. Here, we fabricated and characterized a novel durable-polymer drug-eluting 316 L stainless steel (SS) stent, in which the inner surface was coated with a Ti–O film using the magnetron sputtering method to promote the growth of endothelial cells (ECs). On the outer layer of the stent, first, a Ti–O film was deposited and, then, on top of it a rapamycin-loaded PTMC coat was deposited using the ultrasonic atomization spray method. This dual coating inhibited the migration and expansion of smooth muscle cells (SMCs). The drug coating also inhibited the adhesion/activation of platelets. In tests on dogs, it was found the novel stent promoted re-endothelialization and reduced restenosis, in contrast to the plain SS stent. Thus, the novel stent may have promise for use in treating patients with CAD.

## 1. Introduction

**Figure 1:**
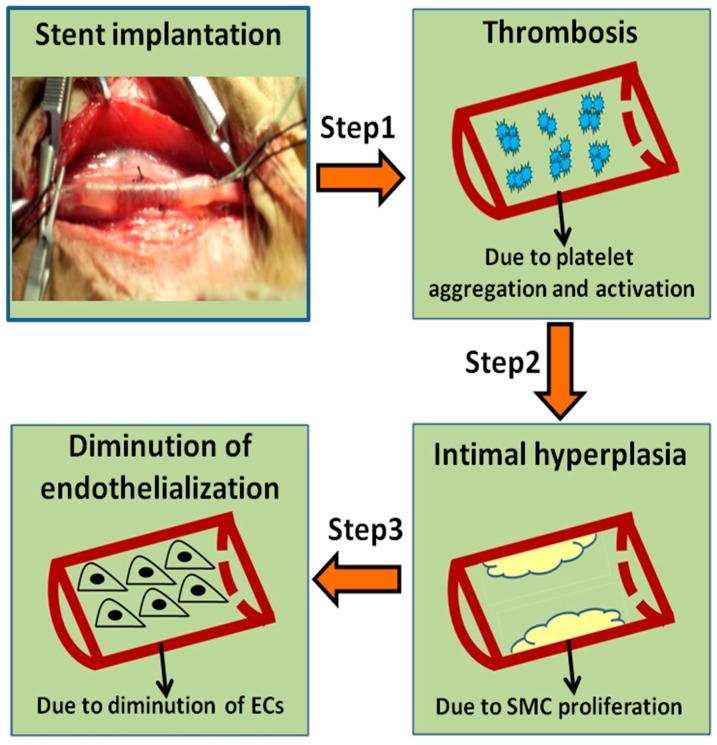
The programmed processes are needed after stent implantation.

Coronary artery diseases (CADs) are one of the major causes of human death and disability in the world [1,2]. Percutaneous coronary intervention (PCI) is considered to be one of the most effective ways to treat CADs. When stents are implanted into the injury site, a series of programmed processes will take place in sequence, with an order of thrombosis, smooth muscle cell (SMC) proliferation, and endothelialization, respectively (Figure 1) [3]. Percutaneous transluminal coronary angioplasty (PTCA) and bare metal stents (BMS) are applied for relatively early treatments, but restenosis rate reaches 30–40% [4,5]. Recently, many strategies have been reported to improve cure efficiency, such as drug-eluting stents (DES) [6], stent-based gene therapy [7], endothelial progenitor cell-capture stents (EPC) [8], bioresorbable scaffolds (BRS) [9] and so on. Although these stents played a very important role and showed promising prospects in the treatment of restenosis, today DES is still the major treatment options for CADs in the clinic, which can significantly decrease the occurrence rate of restenosis to 3–20% [10]. However, drugs such as rapamycin and paclitaxel, etc. coated on DES usually have no selectivity in inhibiting SMCs and ECs, causing the delay of re-endothelialization. Therefore, an ideal DES should have dual functions, not only to inhibit the proliferation of SMCs but also to promote the growth of ECs.

Polymers DES can be divided into nondegradable and degradable polymer-based coatings. The nondegradable polymer coatings are replaced by degradable polymers, due to the fact that permanent foreign bodies in vivo can lead to inflammatory responses and delayed healing [11]. Degradable polymers, especially polylactic acid (PLA), polyglycolic acid (PGA) and their copolymers, are the most frequently applied coating materials for the stent. However, the above polymers cause bulk erosion, causing problems such as poor mechanical performance and acidic products generated from polymer degradation. They may lead to inflammatory responses and induce neointimal hyperplasia and thrombosis [12,13,14,15,16]. Poly (1,3-trimethylene carbonate) (PTMC) as surface erosion material is approved by the Food and Drug Administration (FDA). It can maintain a uniform surface and has no acidic products generated during the degradation process. It has high original mechanical strength and low platelets adhesion and activation [17,18,19,20]. Hence, PTMC was selected as a degradable polymer coating for DES in this study. Rapamycin (also called Sirolimus) is a macrolide immunosuppressant extensively applied in DES and has an obvious effect in inhibiting the migration and proliferation of smooth muscle cells (SMCs), thus reducing restenosis [21,22]. Here, we chose the PTMC/rapamycinas drug and its polymer carrier of the DES.

Generally, stainless steel (SS) stents have some problems to be resolved for better biomedical applications. Allergic reactions can be induced by metal ions such as nickel, chromium, and molybdenum from316L stainless steel (SS) stents. In addition, SS stents suffer from high stenosis rate even have fatal outcomes [23,24,25]. The Ti–O film possesses advantages of good mechanical and chemical durability, reduced platelet adhesion and activation, and accelerated endothelialization in vivo [26,27,28,29]. Hence, in order to improve the performance of stents, the Ti–O film was chosen as SS stent coating in this paper.

The following were the purposes of the study: (i) Determination of in vitro degradation behavior and surface properties of PTMC; (ii) fabrication of a stent from 316 stainless steel only (SS), deposition of a Ti–O film on the inner surface of the SS stent, preparation of PTMC, preparation of a rapamycin-loaded PTMC coating, and deposition of, first, a Ti–O film, and, then, a rapamycin-loaded coating on the outer surface of the SS stent (novel stent); and (iii) determination of in vitro adhesion and activation of human blood platelets, adhesion of human endothelial cells, and adhesion of human smooth muscle cells on SS stent, Ti–O, PTMC, and novel stent.

## 2. Materials and Methods

### 2.1. Materials

Rapamycin was purchased from North China Pharmaceutical Group New Drug Research and Development Co., Ltd. Poly (1,3-trimethylene carbonate) (PTMC, Mn = 500,000, named as P50) and poly (lactic-co-glycolic acid) (PLGA, Mn = 100,000, lactic acid:glycolic acid = 85:15) were acquired from Jinan Daigang Biomaterial Co., Ltd. (Jinan, Shandong, China). P50 polymers with 5 wt.%, 20 wt.%, and 42 wt.% rapamycin were coded as P50R5, P50R20, and P50R42. Dulbecco’s modified eagle medium/Ham F12 medium (DMEM/F12), M199 medium, and fetal bovine serum (FBS) were obtained from HyClone company (Logan, Utah, USA). MTT (3-(4,5-dimethylthiazol-2-yl)-2,5-diphenyl tetrazolium bromide) was purchased from Amresco Inc. (Solon, Ohio, USA). All the chemicals were reagent grade.

### 2.2. In Vitro Degradation of Poly (1,3-trimethylene carbonate)

The in vitro degradation behavior of PTMC polymer was investigated. Pre-weighted PTMC was immersed and incubated in 40 mL phosphate buffer saline (PBS) (pH 7.4) at 37 °C and 80 rpm. Then, lipase (3000 U/L) was added to the PBS solution. Five parallel samples were recovered at predetermined intervals, then rinsed with distilled water to remove residual buffer salts and dried to constant weight in vacuum desiccators. The samples were weighed by an electronic balance (Sartorius ME5, Gottingen, Germany). The mass loss was determined by comparing the dry weight remained at a specific time to the initial weight. Then, the molecular weight of recovered matrix polymer was determined by gel permeation chromatography (GPC waters 2695 and 2414, Milford, MA, USA). The surface morphology of polymers after degradation was also studied by scanning electron microscopy (SEM, Quanta200, Philips, Amsterdam, Netherlands).

### 2.3. Fourier Transform Infrared

The structure of rapamycin, PTMC, and rapamycin-loaded PTMC films was examined with a Fourier transform infrared (FTIR, Thermo Nicolet 5700, Waltham, Massachusetts, USA) instrument. The scanning FTIR range was 4000–400 cm^−1^.

### 2.4. Contact Angle Tests

In contact angle measurement, purified water was dropped on the surface of SS, Ti–O, P50, P50R5, P50R20, and P50R45 by using a microsyringe attached on the goniometer. At least 6 droplets were tested on different parts of each sample (*n* = 3). The contact angles were measured by a Krüss GmbH DSA 100 Mk 2 goniometer (Hamburg, Germany).

### 2.5. Fabrication of Rapamycin-Loaded PTMC Coatings

The PTMC coatings were prepared via casting method. The casting solution was obtained by dissolving PTMC in dichloromethane. The solution was then slowly poured into cleaned glass Petri dishes for obtaining coatings. The coatings were allowed to slowly evaporate the solvent for 48 h and then kept in vacuum for evaporating the residual solvent. Rapamycin-loaded PTMC coatings were prepared in the same way by dissolving 5%, 20%, and 42% (w/w) rapamycin with PTMC in dichloromethane coded as P50R5, P50R20, and P50R42, respectively. Especially for the stent expansion test, spray solution was prepared by dissolving rapamycin and PTMC in dichloromethane and sprayed onto the stent surface.

### 2.6. Fabrication and Surface Morphology Study of Rapamycin-Eluting PTMC Stent

The 316 stainless steel tube was incised into a stent with the help of a laser cutting machine. The stents were carefully polished using the electrochemical method, then cleaned successively with acetone, ethyl alcohol, and distilled water under the condition of ultrasound. Then, the stents were kept under vacuum for evaporating the residual water. Stainless steel stents were coated with the Ti–O film using the magnetron sputtering method. Then the spray solution was prepared by dissolving rapamycin and PTMC in dichloromethane and sprayed onto the outer layer of the stent. The stent was placed on a mandrel to prevent drug leaking onto the luminal stent surface during spray coating. The whole system was controlled by a computer.

The surface morphology of the rapamycin-eluting stent after the expansion was observed by scanning electron microscopy (SEM, Quanta 200, Philips, Amsterdam, Netherlands). The stent was mounted onto the angioplasty balloon and dilated at the pressure of 4.0 atm.

### 2.7. In Vitro Platelets Adhesion Test

Platelet-rich plasma (PRP) was obtained by centrifuging fresh human whole blood containing 3.8 wt.% citrate acid at 1500 rpm for 15 min. Then, the SS, Ti–O, P50, P50R5, P50R20, and P50R42 samples were immersed in 0.5 mL PRP individually, and incubated at 37 °C for 45 min. Next, the samples were rinsed with PBS three times to remove the weakly adherent platelets, and the adherent platelets were fixed in 2.5% glutaraldehyde solution for 12 h. After the treatment of dehydrating, dealcoholizing, and critical point drying, the samples were sputter-coated with gold and imaged by scanning electron microscopy (SEM, Quanta200, Philips, Amsterdam, Netherlands).

### 2.8. Platelet Activation Evaluation

P-selection expressing (also called GMP140): Fresh whole blood was centrifuged for 15 min at 1500 rpm to obtain PRP for p-selectin expressing. P-selection expression in plasma was determined using enzyme-linked immunosorbent assay. The samples in a 24-well culture plate were incubated in PRP for 45 min at 37 °C and then rinsed with calf serum albumin PBS solution three times. Subsequently, these samples were shifted into a new 24-well culture plate and injected with 200 μL rat anti-human CD62P antibody into each well for incubating 1 h at 37 °C. Then, 200 μL horseradish peroxide–enzyme goat anti-rat polyclonal antibody was added into each well. After being cultured for 1 h at 37 °C, samples were rinsed using PBS solution and then transferred into a new 24-well culture plate, and then 140 μL TMB solution was injected into each well. After reaction for 10 min, 50 μL of 1 M H_2_SO_4_ was added to stop the reaction, and 160 μL of each mixed solution was transferred into a 96-well plate, and the absorbance was read at 450 nm.

Lactate dehydrogenase (LDH) assay: The samples were placed into a 24-well plate, then PRP (100 μL) was added onto each sample surface. After being incubated at 37 °C for 45 min, each sample was rinsed with PBS three times. Following that, 40 μL of Triton-X-100 (diluted to 1%) was added onto the surface. Then, 5 min later, 25 μL of the lysates was taken from the surface and mixed with the 200 μL substrate solution containing nicotinamide adenine dinucleotide-reduced and sodium pyruvate in the wells of a 96-well plate. A calibration curve was constructed, and the slope of the curve was evaluated and calibrated with lysates of all platelets at the absorbency of 340 nm.

Fibrinogen denaturation: The fibrinogen adsorption was analyzed by incubating the specimen with 50 μL of platelet-poor plasma (PPP) at 37 °C for 2 h. The activation of the adsorbed fibrinogen was determined by the exposure of the γ chain via indirect immunochemistry using a sensitive primary antibody (Accurate Chemical).

### 2.9. Endothelial Cells Assay

Human umbilical vein endothelial cells (ECs) were obtained from the umbilical arteries of newborns kindly provided by the Women and Children Healthcare Centre of Chengdu. ECs were cultured in M199 medium containing 10% FBS in a humidified atmosphere of 95% air and 5% CO_2_ at 37 °C. Culture medium was changed every two days and cells were passaged by trypsinization. After being cultured for 1 day, samples were washed three times with PBS, fixed in 2.5% glutaraldehyde solution for 12 h. After the treatment of dehydrating, dealcoholizing, and critical point drying, the samples were sputter-coated with gold and imaged by scanning electron microscopy (SEM, Quanta 200, Philips, Amsterdam, Netherlands).

### 2.10. Smooth Muscle Cell Culture

Human primary smooth muscle cells (SMCs) were obtained from umbilical arteries of newborns kindly provided by the Women and Children Healthcare Centre of Chengdu. SMCs were cultured in DMEM/F12 medium containing 10% FBS in a humidified atmosphere of 95% air and 5% CO_2_ at 37 °C. Culture medium was changed every two days and cells were passaged by trypsinization.

### 2.11. Smooth Muscle Cellss Staining Evaluation

Cells at passages 3 were selected for the experiments. Cells were plated onto different sample surfaces at an initial density of 1 × 10^5^ cells/mL and were separately cultured on samples for 1, 3, and 5 days, washed three times with PBS, and then fixed with 2.5% glutaraldehyde for 12 h. In order to stain cytoplasm and nuclear, we adopted staining agents of α-smooth muscle actin (α-SMA) and 4’,6-diamidino-2-phenylindole (DAPI). SS, Ti–O, P50R5, P50R20, and P50R42 samples were washed three times with PBS to remove glutaraldehyde. Cells were then incubated for 60 min with a monoclonal rabbit anti-human actin antibody, and samples were washed three times with PBS. Next, the samples were incubated for 30 min with biotinylated goat anti-rabbit IgG and washed three times for 2 min each with PBS. Following that, DAPI was added and incubated at room temperature for 5 min, and samples were washed five times with PBS. Finally, the cells were viewed and photographed using a fluorescence microscope (DMRX, Leica, Leitz, Wetzlar, Germany).

### 2.12. 3-(4,5-dimethyl-2-thiazolyl)-2,5-diphenyl-2-H-tetrazolium bromide Assay

The diameter of samples was 10 mm; cells were planted on the sample surface in the cell plates. After culture for 1, 3, and 5 days, 100 μL of the 3-(4,5-dimethyl-2-thiazolyl)-2,5-diphenyl-2-H-tetrazolium bromide (MTT) solution was added to all the sample wells, and the plate was incubated for 4 h at 37 °C in a humidified atmosphere of 5% CO_2_. At the end of the incubation period, the medium was removed and 400 μL of dimethylsulfoxide (DMSO) was added to each sample in the 24-well plate to dissolve the formazan crystals; 200 μL of MTT solution of the cells was then transferred into a 96-well plate, and the absorbance of the samples was measured at 490 nm.

### 2.13. Animal Study

All procedures were in compliance with the China Council on Animal Care and Southwest Jiaotong University Laboratory Animal Ethics Committee and followed all the ethical guidelines for experimental animals. In this work, 4 dogs weighing about 15 kg and 8 stents were used. The dogs were divided into two groups: The SS stent as control and the P50R42 stent (*n* = 4 stents per group). The results of 69 days were evaluated after stents were respectively implanted bilaterally in femoral arteries of dogs. The stents were fixed with 10% formaldehyde in PBS and used for tissue slicing analysis. The cross-sectional slices were stained with toluidine blue method and then used for histomorphometric analysis.

### 2.14. Statistical Analysis

The experiment data collected in this paper were expressed as mean ± standard deviation (SD). The statistical significance of differences between groups was carried out using one-way ANOVA followed by post-hoc analysis. Significance was established by a value of *p* < 0.05, ** *p* < 0.01, ** *p* < 0.001.

## 3. Results

### 3.1. In Vitro Degradation of Poly (1,3-trimethylene carbonate)

**Figure 2:**
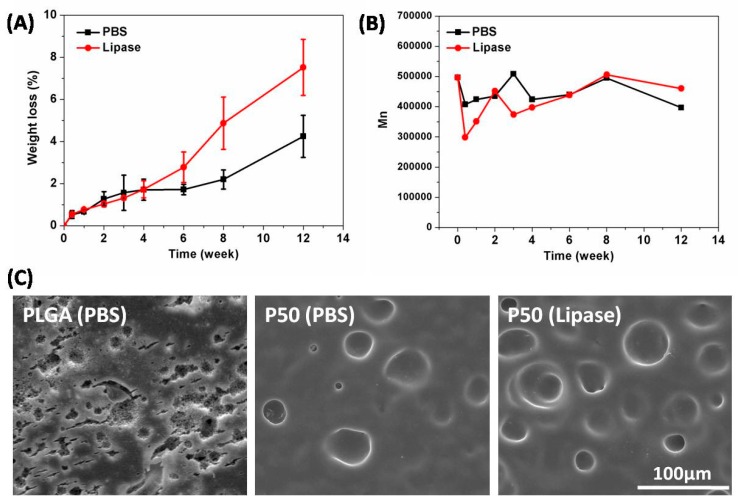
(**A**) Mass loss and (**B**) molecular change of poly (1, 3-trimethylene carbonate) (PTMC) degraded in (PBS) and Lipase (3000 U/L) at 37 °C for different degradation time. (**C**) Surface morphology of poly (lactic-co-glycolic acid) (PLGA) and P50 (PTMC, Mn = 500,000) after being degraded in PBS or lipase for 12 weeks.

We adopted two kinds of liquids, such as PBS and lipase, to evaluate degradation behavior of PTMC film. As shown in Figure 2A, the mass loss of PTMC film was 1.7 ± 0.5% in PBS and 1.7 ± 0.4% in lipase at 4 weeks; lipase did not show advantages compared with PBS at this stage. However, with the increase of degradation time, the degradation rate of PTMC film in lipase was about 2 times faster than that in PBS. The mass loss of PTMC film was 2.2 ± 0.5% in PBS and 4.9 ± 1.2% in lipase at 8 weeks. The mass loss of PTMC film was 4.2 ± 1.0% in PBS and 7.5 ± 1.3% in lipase at 12 weeks. The GPC results indicated that the molecular weight of PTMC remained close to the initial value regardless of whether in PBS or lipase media (Figure 2B). At 12 weeks, surface morphology results demonstrated that it only degraded on the surface of PTMC; the degradation rate of PTMC film in lipase was faster than that in PBS. Nevertheless, the surface and internal degradation of PLGA occurred at the same time (Figure 2C). Therefore, SEM results further showed that PTMC led to surface erosion and PLGA to bulk erosion.

Zhu et al. was the first person to research the properties and biodegradation of PTMC [30]; since then, other researchers have extensively studied the degradation of PTMC. In our work, we have proven that PTMC film in lipase degrades significantly faster than that in PBS. Because PTMC was a hydrophobic material, it has been reported that lipase activity can be enhanced on the hydrophobic surface. Similar results were reported by other groups; the molecular weight of PTMC kept stable during degradation in vitro. Several studies have shown that the degradation rate of PTMC (329 kDa, losing 50.9% after 12 weeks) was faster than that in our work (500 kDa, losing 7.5% after 12 weeks). We considered the reasons as follows: (i) Different sources of lipase: We used porcine pancreas lipase and researchers used lipase from thermomyces lanuginosus; (ii) different lipase concentrations: We adopted 3000 U/L lipase solution, but literature did not show details about the concentration of lipase; (iii) the sample size and molecular weight are different. In a word, PTMC possesses a higher degradation rate in lipase and presented surface erosion characteristics [31,32].

### 3.2. Characteristics and Morphology

**Figure 3:**
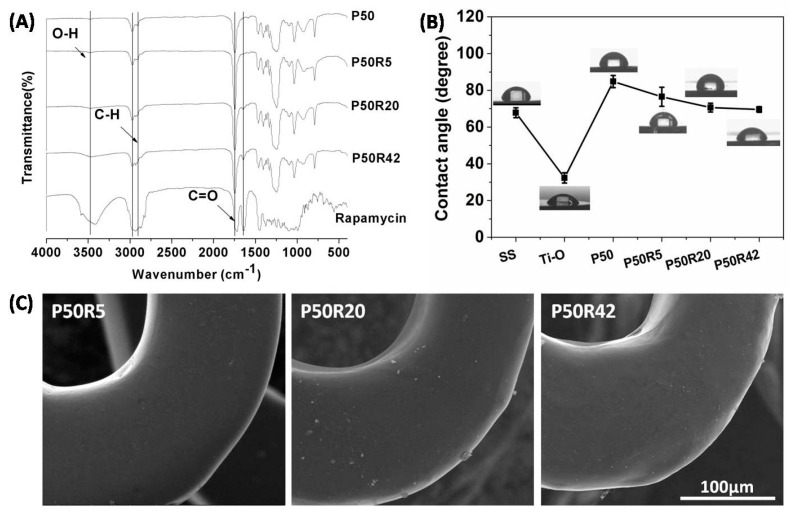
(**A**) FTIR spectra of rapamycin, P50, P50R5, P50R20, and P50R42. (**B**) Contact angles of stainless steel (SS), Ti–O, P50, P50R5, P50R20, and P50R42. (**C**) SEM images of P50R5, P50R20, and P50R42 stents after post-expansion.

The chemical structure of PTMC/rapamycin coating was evaluated using FTIR. As shown in Figure 3A, for the P50 polymer, 2970 cm^−1^ and 2918 cm^−1^ was C–H stretching vibration peak, 1746 cm^−1^ was C=O stretching vibration peak, C–H flexural vibration peak appeared at 1461 cm^−1^ and 1398 cm^−1^, and 1300 cm^−1^–1000 cm^−1^ was C–O stretching vibration peak and C–H swing peak. In addition to the same peaks as P50, rapamycin also had an O–H stretching vibration peak (3473 cm^−1^) and C=C stretching vibration peak (1637 cm^−1^). After mixing rapamycin into the P50 polymer, the coatings retained all of the peaks from rapamycin and P50. These results showed that rapamycin blended in the P50 polymer still kept its chemical stability. There were no chemical reactions between P50 and rapamycin. Our previous research also proved that tacrolimus blended in PTMC also possessed chemical stability by comparing FTIR spectra [17].

The contact angle evaluates the wettability of material surfaces. The lower contact angle reflects an increased surface hydrophilicity and higher surface energy. The wettability of material surfaces can influence platelet and cell attachment behavior [33]. As shown in Figure 3B, the water contact angle on SS was 67.8 ± 2.7°. Ti–O possessed high hydrophilicity, and the value of the water contact angle was 32.3 ± 2.8°. P50 was a hydrophobic material with a contact angle of 84.8 ± 3.3°, while the contact angles on the P50R5, P50R20, and P50R42 sample surfaces were 76.5 ± 5.2°, 70.6 ± 2.4°, and 69.6 ± 1.5°, respectively. The hydrophilicity was increased with the increase of rapamycin drug content. The improvement of the hydrophilicity may be due to the hydrophilic groups of rapamycin, such as hydroxyls [34].

The surface properties, such as smoothness and no cracking and peeling of the stent surface coatings, are one of the important factors leading to restenosis and side effects [11]. The PTMC/rapamycin-coated stent was expanded for 10 s with a mean inflation pressure of 8 atm. The expanded stents were then imaged by SEM. As shown in Figure 3C, after stent expansion, the surface morphology of P50R5, P50R20, and P50R42 drug stents remained smooth and uniform, no cracks could be found, and no visible delamination of the surface coating was found even after the huge deformation of the metal framework during the expansion process. The PTMC/rapamycin coating seemed to be stably conjoined to the stent surface. Mclaughlin and Joner reported on the condition of polymer on current commercial drug-eluting stents and plasma deposition stent after expansion. There were serious cracks and visible delamination, this may have led to aggravating the incidence of thrombosis, neointimal hyperplasia, and clinical restenosis rates after implantation [35,36]. This result manifested in that PTMC/rapamycin drug-eluting stents by ultrasonic spray coating had an advantage over polymer on current commercial drug-eluting stents and plasma deposition stents.

### 3.3. In Vitro Platelet Adhesion and Activation Evaluation

**Figure 4:**
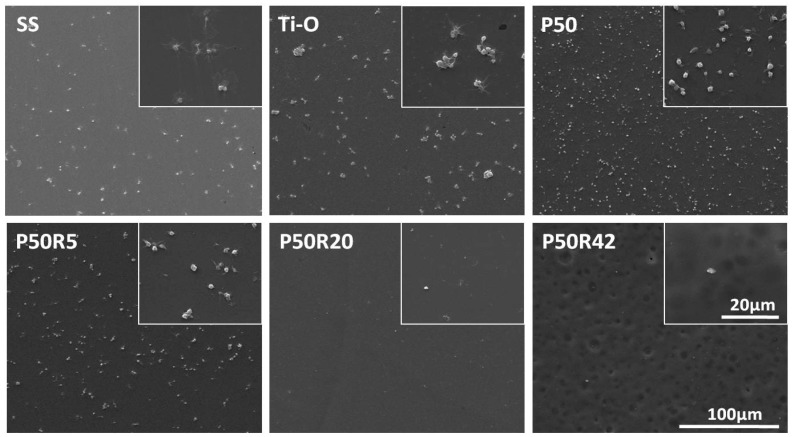
Morphology of adherent platelets on SS, Ti–O, P50, P50R5, P50R20, and P50R42 after 45 min of incubation in platelet-rich plasma (PRP) observed by SEM.

**Figure 5:**
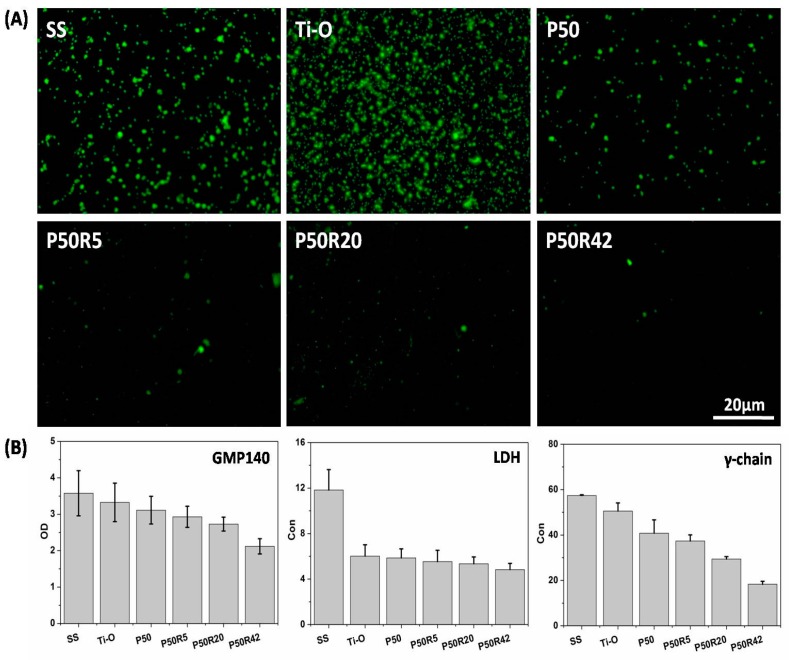
(**A**) P-selectin fluorescent images and (**B**) GMP140, LDH, γ-chain quantitative results of SS, Ti–O, P50, P50R5, P50R20, and P50R42 after 45 min of incubation in PRP.

Platelet adhesion and activation evaluation of drug-polymer films is an important test of the blood compatibility of materials. Here, SS, Ti–O, P50, and drug films were investigated through qualitative and quantitative platelet assays. As shown in Figure 4, platelets were seriously activated, aggregated, and had pseudopodia on the SS surface. On the Ti–O surface, there was still aggregation, but less activation and pseudopodia were found on the Ti–O surface than on the SS surface. There was almost no aggregation on the P50 surface, but a small amount of activation and pseudopodia still existed. When rapamycin was mixed into P50, the amount of platelet adhesion exhibited a sharp decline. There were very few platelets on the P50R5 sample surfaces; platelets can hardly be observed with the increase of drug content on the P50R20 and P50R42 sample surfaces. There was no aggregation, activation, or pseudopodia on these drug sample surfaces. As seen in Figure 5A, P-selectin staining results showed that a large number of this molecules appeared on the SS and Ti–O surfaces, and a small number of molecules existed on the P50 surface. After introducing rapamycin into P50, there were almost no such molecules, indicating that it did not form platelet clots on the P50R5, P50R20, and P50R42 sample surfaces. The results of platelet behavior were further studied by quantitative P-selectin (GMP140), LDH, and fibrinogen denaturation (γ-chain) assays (Figure 5B). From quantitative results, it possessed the most serious platelet activation on SS surface compared with the other samples. Less platelet activation appeared on the Ti–O and P50 samples than on the SS surface. After drugs were added into P50, the values of GMP140, LDH, and γ-chain were obviously lower on the P50R5, P50R20, and P50R42 surfaces than on the SS, Ti–O, and P50 surfaces. The inhibition rate of platelet activation was concentration-dependent. A higher content drugs appeared to lead to the best inhibition. These results illustrated that drug films of P50 and rapamycin played an important role in inhibiting platelet adhesion, activation, and presented good hemocompatibility, and could finally inhibit thrombosis after implanted in vivo. Pan et al. considered that rapamycin could possibly inhibit the deposition of plasma protein and thus decrease platelet adhesion and activation [37].

### 3.4. Endothelial Cells Assay

**Figure 6:**
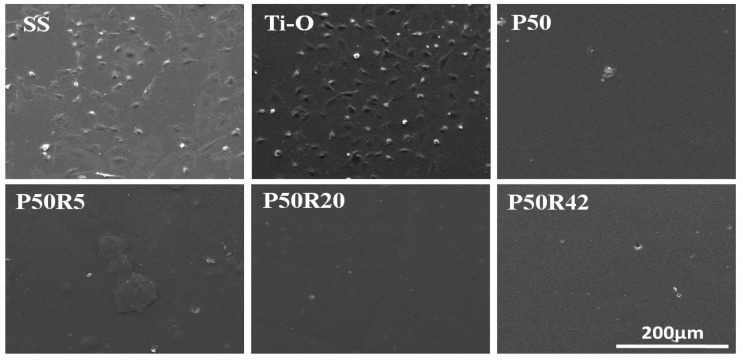
SEM images of endothelial cells after culture on the SS, Ti–O, P50, P50R5, P50R20, and P50R42 sample surfaces for 1 day.

Rapamycin inhibits cells without selectivity. It can prevent the growth of endothelial cells and smooth muscle cells simultaneously [38]. The inhibition mechanism is that rapamycin can combine with FK506 binding protein (FKBP) in cells, thus impeding the transition from the G1 to S stage [39]. As shown in Figure 6, the attachment and morphology of ECs were observed on day 1; ECs preferred to grow on the Ti–O surface rather than on the SS surface. However, there were almost no ECs on the P50R5, P50R20, and P50R42 surfaces. The results demonstrated it is reasonable for us to fabricate stent with a special design. At first, rapamycin was loaded with P50 as the outer layer of the stent in order to inhibit smooth muscle cells and Ti–O film as the inner layer of the stent to promote endothelial cell growth. After drugs were released and proliferation was suppressed, Ti–O films from the inner and the outer layer of stents played a role in re-endothelialization at the same time.

### 3.5. Smooth Muscle Cells Evaluation

**Figure 7:**
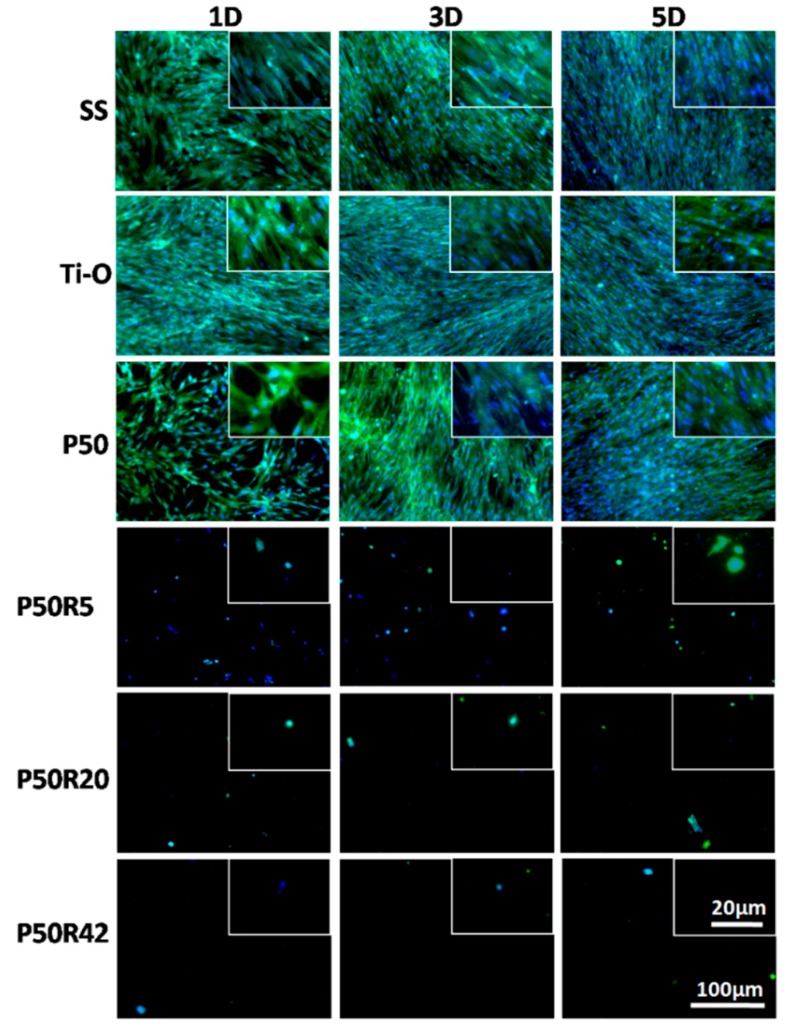
Immunofluorescence staining micrographs of smooth muscle cells (SMCs) actin and DAPI after culture on the SS, Ti–O, P50, P50R5, P50R20, P50R42 sample surfaces for 1, 3, and 5 days.

**Figure 8:**
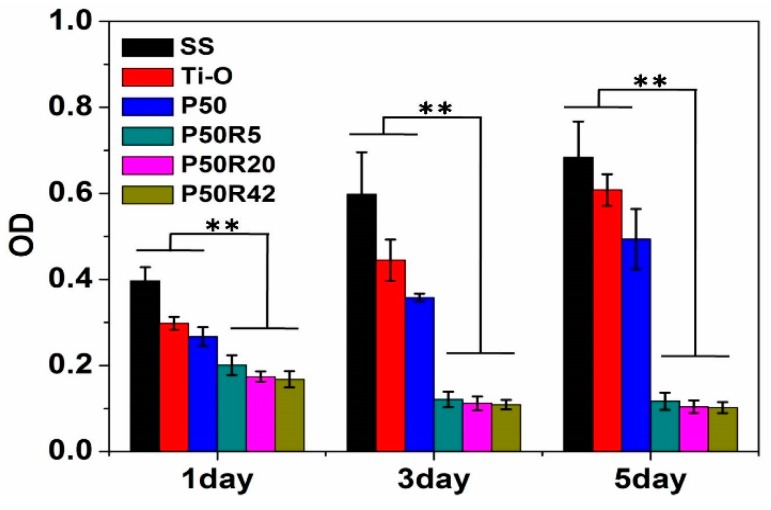
SMC attachment and proliferation cultured on the surfaces of SS, Ti–O, P50, P50R5, P50R20, and P50R42 using MTT (3-(4,5-dimethylthiazol-2-yl)-2,5-diphenyl tetrazolium bromide) assay (** *p* < 0.01).

SMCs play an important role in stent-restenosis and delayed re-endothelialization. A drug-eluting stent can inhibit SMC migration and proliferation at the first stage after implanting the stent in vivo [40]. As shown in Figure 7 and Figure 8, the attachment and proliferation of SMCs were investigated after culture for 1, 3, and 5 days through qualitative (fluorescence) and quantitative (MTT) evaluation of SMCs. The growth behavior of SMCs was observed on the SS, Ti–O, P50, P50R5, P50R20, and P50R42 surfaces through nucleus and cytoplasm staining (Figure 7). SMCs preferred to grow on the SS, Ti–O, and P50 surfaces; these materials could not inhibit the adhesion and proliferation of SMCs. After adding rapamycin into P50, the adhesion and growth ability of SMCs could be obviously suppressed. These results indicated that rapamycin can obviously inhibit the attachment and proliferation of SMCs.

The quantitative proliferation results of SMCs by MTT are shown in Figure 8. There were fewer SMCs on the P50 surface than on the SS and Ti–O surfaces. After introducing the drug of rapamycin, the proliferation of SMCs was obviously inhibited on the P50R5, P50R20, and P50R42 surfaces. The proliferation trend of SMCs by MTT and staining was consistent. Thus, the above results demonstrated that PTMC/rapamycin coating stent could inhibit the adhesion and proliferation of SMCs, which indicated that rapamycin may contribute to reducing intimal hyperplasia and inhibit restenosis after stent implantation.

### 3.6. Animal Study

**Figure 9:**
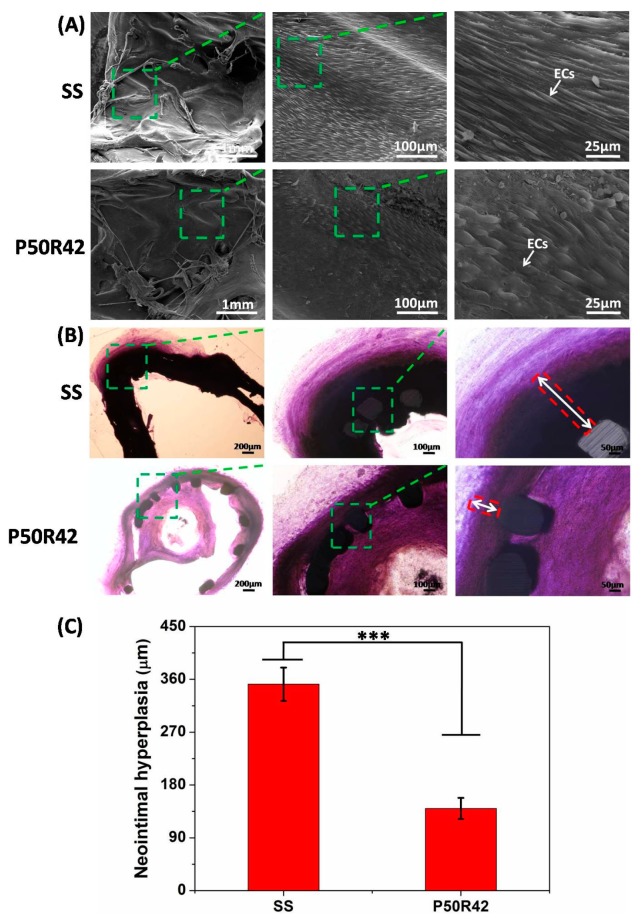
The results of control SS- and P50R42-coated stents after being implanted bilaterally in a dog’s femoral arteries for 69 days assessed by (**A**) SEM and (**B**) histomorphometric analysis. (**C**) The thicknesses of neointimal hyperplasia on control SS and P50T42 stents (*** *p* < 0.001).

In this study, the in vivo implantation of SS and P50R42 stents in femoral arteries of dogs was harvested at 69 days. An ideal drug stent should increase endothelial regeneration from the inside of the stent and inhibit intimal hyperplasia to reduce restenosis from outside of the stent. As shown in Figure 9A, the SEM images indicated that the surface of SS was fully covered by ECs, and the EC monolayer also almost fully covered the P50R42 stent surface. Histomorphometric analysis was further carried out in order to evaluate the effect of stents on restenosis. Figure 9B showed the toluidine blue method stained images of cross-sections of arteries with stents. The stained images demonstrated that the P50R42 stent significantly reduced neointimal hyperplasia compared to SS stent. In addition, the thicknesses of neointimal hyperplasia were 140.0 ± 18.0 μm on the P50R42 stent and 351.7 ± 28.4 μm on the SS stent, respectively (Figure 9C). These results showed that the degree of intimal hyperplasia was reduced by 60% on P50R42 stent compared to the SS stent. Therefore, both SS and P50R42 stents were beneficial to reendothelialization. However, the intimal hyperplasia of SS stent was very serious. By contrast, P50R42 presented an excellent performance in inhibiting intimal hyperplasia. Herein, the in vivo results are strongly consistent with the in vitro results, and they are in agreement with our design concept. Although we adopted an exquisite design to fabricate the stent’s outer and inner layer, it is still limited in promoting endothelial cell growth for Ti–O coating as the inner layer. Therefore, it may be an effective way to accelerate endothelialization by endowing function on Ti–O film, such as introducing a protein and growth factor. We are going to explore these areas in our following studies.

## 4. Discussion

When drug-eluting stents (DES) as a foreign body are implanted into the lesion site, this process will inevitably lead to endothelial denudation and damage. Then, ECs and platelets will be activated, thus easily causing acute thrombus formation [41]. The monocytes can be recruited to a stent implantation site when chemokines are released from activated ECs and platelets. Therefore, the early inflammation response will happen and accompany macrophage infiltration [42,43]. Subsequently, platelet activation and subacute thrombosis can be accelerated by inflammation reaction, then causing SMCs’ excessive proliferation. The phenomena will result in intimal hyperplasia and restenosis [3,44]. Several studies have shown that this is the key to inhibiting the rapid proliferation of SMCs after stent implantation for 1–2 weeks [45]. Hence, the thrombosis, inflammation, and SMC proliferation should be effectively inhibited within 2 weeks after implanting drug stents.

In this work, we designed PTMC/rapamycin drug coating as the outer layer of stents in order to inhibit SMC proliferation at an early stage. In vitro experiments indicated that PTMC/rapamycin drug films with 5 wt.%, 20 wt.%, and 42 wt.% rapamycin can inhibit the adhesion and proliferation of SMCs (Figure 7 and Figure 8), thus presenting antirestenosis properties. Moreover, PTMC/rapamycin drug films can also inhibit platelet adhesion and activation through qualitative (adhesion) and quantitative (GMP140, LDH, and γ-chain) assays, thus indicating excellent anticoagulant properties and possessing the antithrombotic ability. Although we did not investigate inflammatory reaction in this study, some investigators have shown that rapamycin can inhibit macrophage adhesion and reduce the release of pro-inflammatory factors IL-1β and IL-6, thus presenting anti-inflammatory properties as well [46]. Therefore, PTMC/rapamycin drug coating as the outer layer of stents exhibited excellent inhibition effects on SMC proliferation, thrombosis, and inflammation.

Although rapamycin can inhibit the proliferation of SMCs and further prevent intimal hyperplasia, it can also inhibit EC growth and prevent intimal repair at the same time, leading to the delayed endothelialization. This is the major factor to lead to in-stent thrombosis at the middle stage from 1 to 3 months [47]. Thus, in this study, we fabricated Ti–O film coating as the inner layer of stents to promote EC growth and to facilitate endothelialization, preventing middle-to-late thrombosis and restenosis. Figure 6 illustrates that Ti–O film was indeed beneficial to EC growth. It is also demonstrated that Ti–O film was superior to SS in inhibiting platelet adhesion and activation, as shown in Figure 4 and Figure 5. Furthermore, the in vivo results also demonstrated that Ti–O film is beneficial to re-endothelialization and PTMC/rapamycin drug coating could inhibit intimal hyperplasia (Figure 9). Therefore, based on the natural structure of blood vessels and the pathological response after stent implantation, PTMC/rapamycin drug coating as the outer layer and Ti–O film as the inner layer of stents was designed, which not only can inhibit thrombosis, inflammation, and SMC proliferation, but can also promote endothelialization at the same time. This kind of drug stents is a better choice compared to existing DES and may possess potential clinical applications.

## 5. Conclusions

In this study, surface-erosion PTMC instead of bulk-erosion PLA or PLGA was chosen as the rapamycin carrier. The functional stents include three layers: 316L stainless steel stents, Ti–O film, and PTMC/rapamycin drug coatings. PTMC/rapamycin drug coatings were sprayed on one side of the stents facing the vascular surface to inhibit smooth muscle cells from the outer layer, and Ti–O film promoted endothelial cells from the inner layer. The related evaluation of platelet adhesion, GMP140, LDH, and γ-chain assays indicated that PTMC/rapamycin drug coating can hinder platelet adhesion, activation, and aggregation. The rapamycin drug can inhibit the growth of SMCs and ECs. However, Ti–O film is beneficial for endothelial cell growth. Therefore, it is advantageous to fabricate the stent with two layers inside (SS and Ti–O) and three layers outside (SS, Ti–O, and PTMC/rapamycin). Choosing surface-eroding polymer PTMC and designing functional stents in this study may have potential applications as a vascular stent device. When the drug was released and the proliferation was suppressed, the Ti–O films simultaneously promoted the re-endothelialization of the stents. The PTMC/rapamycin drug coating was uniform, with no cracking or peeling found after stent expansion. The in vitro results showed that Ti–O film promoted endothelial cell growth and the drug coating possessed obvious advantages in inhibiting adhesion/activation of platelets, and the attachment and proliferation of SMCs. Meanwhile, the in vivo results indicated that PTMC/rapamycin drug stent can promote re-endothelialization from the inside of the stent and significantly reduced neointimal hyperplasia from the outside of the stent. The degree of intimal hyperplasia was reduced by 60% on P50R42 stent compared with the SS stent. Thus, our novel stent may be promising for treating patients who have CAD.
